# Data for the description of fungal diseases and agronomic parameters of Mango ginger (*Curcuma amada* Roxb.)

**DOI:** 10.1016/j.dib.2018.11.065

**Published:** 2018-11-16

**Authors:** Victor O. Ayodele

**Affiliations:** Department of Pure and Applied Botany, College of Bio-Sciences, Federal University of Agriculture Abeokuta, PMB 2240 Abeokuta, Nigeria

## Abstract

This data article contains data, related to fungal diseases of Mango ginger (*Curcuma amada* Roxb.), that were collected at Federal University of Agriculture Abeokuta. Pictures described leaf spot, leaf blight and rhizome rot diseases, and associated fungi and fungus-like organisms were listed. Data of plant height and disease incidence, against plant age was described with graphs. Further, data of disease severity for planting years of 2016 and 2017 were compared and percentage commercial loss of rhizome rot for the planting years calculated https://doi.org/10.1016/j.cpb.2018.10.001 (Ayodele et al., 2018).

**Specifications table**

**Table:** 

Subject area	Plant Pathology
More specific subject area	Plant Diseases
Type of data	Tables
How data was acquired	Survey, Digital Camera, Microscope
Data format	Raw, analyzed
Experimental factors	Diseased samples taken to the laboratory were rinsed with tap water
Experimental features	Survey Plantings, Samples Collection at Survey Plots, Isolation and Identification of Fungi and Fungus-Like Organisms
Data source location	Abeokuta, Nigeria and/Latitude 7.216463,Longitude 3.441747; Latitude 7.215908, Longitude 3.4238338
Data accessibility	Data is with this article
Related research article	Ayodele VO, Olowe OM, Afolabi CG, Kehinde IA, Identification, Assessment of Diseases and Agronomic Parameters of Curcuma amada Roxb (Mango ginger), Current Plant Biology (2018), https://doi.org/10.1016/j.cpb.2018.10.001

**Value of the data**

• The data can be used as information about the diseases to which *Curcuma amada* could be susceptible to.
• It could also be used to forecast epidemic about the cultivation of *C. amada*.
• Data may be used to give direction for disease management of *C. amada*.

## 1 Data

The picture data describes the various disease symptoms observed with Mango ginger.

**Fig. 1:**
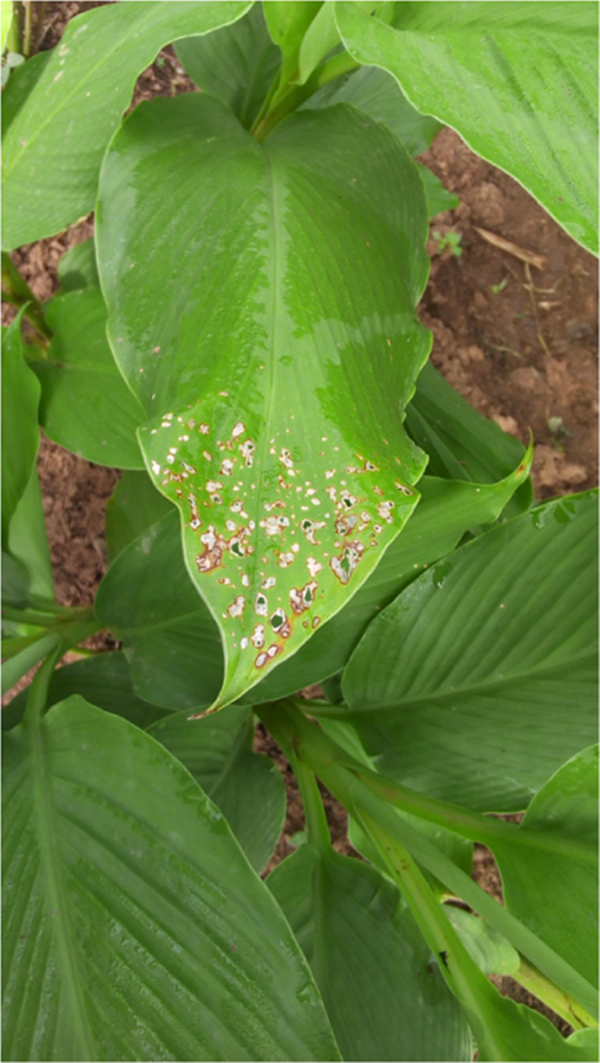
: Mango ginger leaf being eaten up with leaf spot.

Leaf Spot: It started with a visible small white spot, usually with yellow or brown halo. Spot may expand or multiply on leaf to later varnish into holes and shreds in the affected leaf (Fig. 1).

**Fig. 2:**
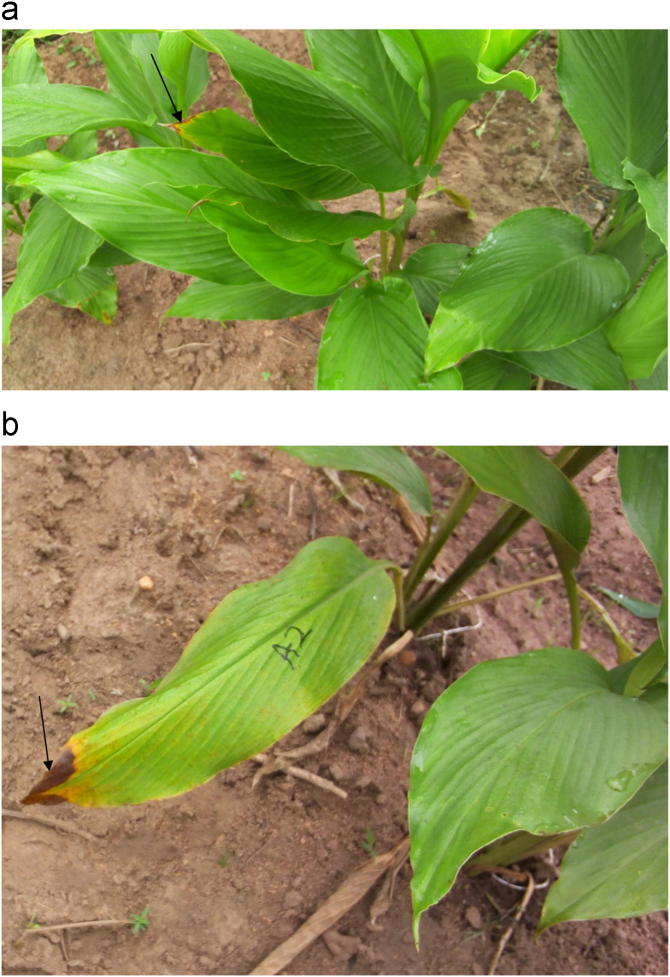
(a) Arrow points early blighting stage of leaf: Yellowing, then brown lesions. (b) Arrow points symptom of a progressive leaf blight disease of Mango ginger. (For interpretation of the references to color in this figure, the reader is referred to the web version of this article).

Leaf Blight: Yellow lesion on blade, leaf edge or tip turned brown and proliferates to surrounding healthy tissues killing them (Fig. 2).

**Fig. 3:**
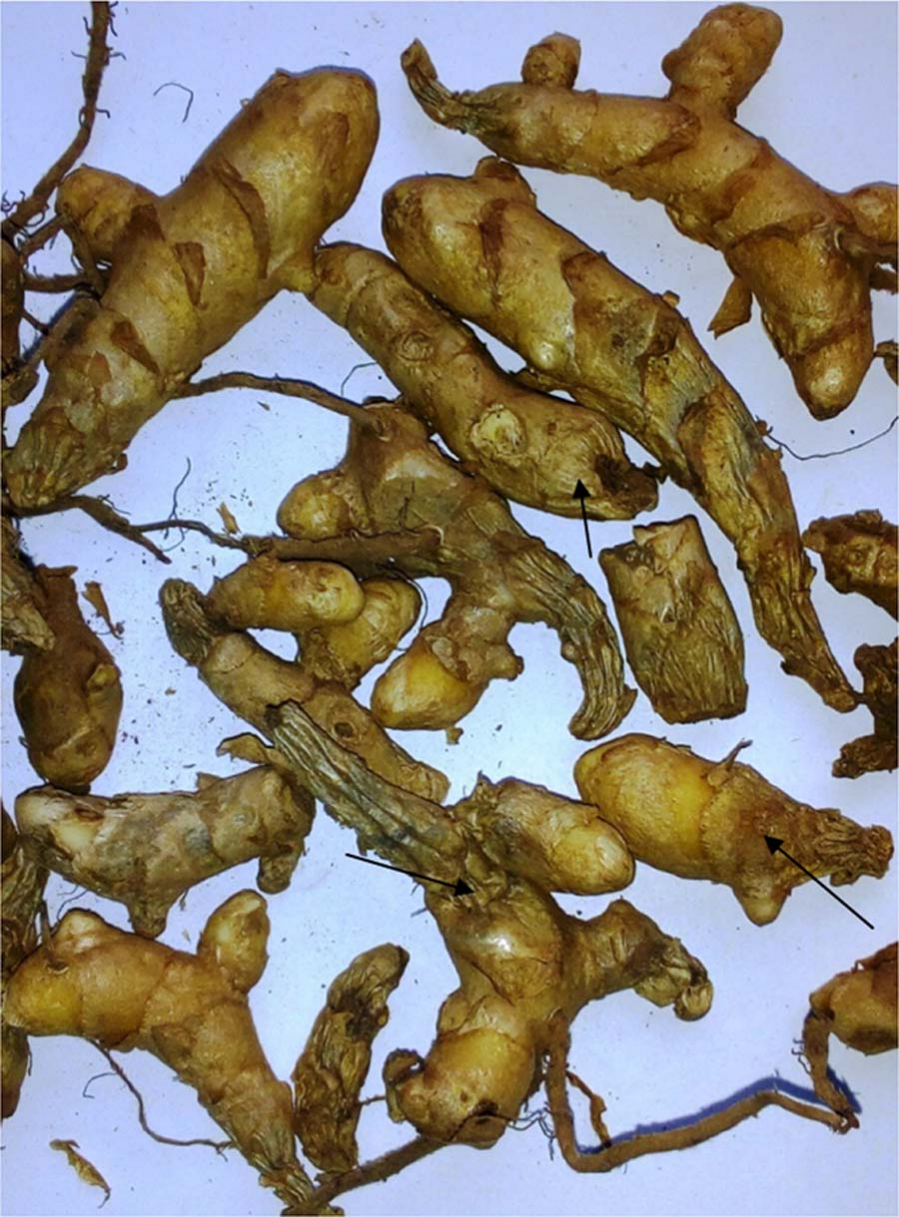
Sorted rhizomes affected with rots.

Rhizome rot: Infected rhizomes felt soft and water soaked with foul odour (unlike the characteristic smell of raw mango) and later became dry (Fig. 3); (Table 1).

**Table 1:** Frequently Isolated fungal pathogens associated with the leaves and stems of *Curcuma amada* plants.

Name of pathogen	Associated disease
*Alternaria alternata*	Leaf blight
*Colletotrichum gloeosporioides*	Leaf blight
*Colletotrichum capsici*	Leaf spot
*Phyllosticta zingiberi*	Leaf spot
*Cercospora curcumae*	Leaf spot
*Rhizoctonia solani*	Rhizome rot/Leaf blight
*Pythium aphanidermatum*	Rhizome rot
*Fusarium solani*	Rhizome rot

The data for plant height, number of leaves, disease incidences and severities were taken concurrently on weekly base for 24 weeks (Table 2).

**Table 2:** Raw data of plant height (PH), number of leaves (NL), spot disease incidence (SDI), blight disease incidence (BDI), spot disease severity (SDS) and blight disease severity (BDS) for year 2016 and 2017.

Week from emergence	PH 2016	PH 2017	NL 2016	NL 2017	SDI 2016	SDI 2017	BDI 2016	BDI 2017	SDS 2016	SDS 2017	BDS 2016	BDS 2017	
1	2.6	1.2	1	1	0.32	0	0	0	2.3	0	0	0	
2	4.4	3.8	2	2	0.49	2.4	0	0	5.8	0	0	0	
3	8.2	7.8	3	3	0.73	3.7	0	0	12.3	6.3	0	0	
4	15.9	14.5	3	4	1.15	5.7	0	0	12.9	9.5	0	0	
5	17.5	21	4	5	3	3.7	0.38	0	25.1	14.3	2.9	0	
6	23.3	26.9	4	5	4.3	13.4	0.84	0	29.8	17.5	5.8	0	
7	28.9	30.4	5	6	5.06	8.1	1.76	0	31	23.8	12.9	0	
8	34.7	33	6	7	5.8	11.4	5	0	28.7	30.2	17	0	
9	39.8	36.9	6	7	10.5	10.2	5.6	12.2	22.8	39.7	20.5	0	
10	43.5	41.6	7	8	9.8	12.2	6.3	12.6	19.3	36.5	24	11.1	
11	46.1	45.6	7	8	11	8.5	6.9	13	18.1	39.7	28.1	11.1	
12	49.4	50.2	8	9	12.1	2	9.7	11.8	18.1	25.4	40.4	11.1	
13	54.7	54.6	8	9	4	5.7	5.5	20.3	18.1	27	42.7	20.6	
14	60.5	59.4	9	8	4.3	6.7	6.6	14.2	20.5	23.8	50.9	23.8	
15	63.1	64.9	8	8	4.6	4.9	8.8	13.4	18.1	23.8	56.7	30.2	
16	66.6	69.6	8	8	4.9	8.5	7.8	17.9	14.6	14.3	70.8	36.5	
17	70.9	74	8	8	5.2	7.3	8.2	19.5	15.8	14.3	76.6		42.9
18	73.4	78.4	7	8	1.8	6.5	7.3	24.8	14.6	14.3	78.9	52.4	
19	75.3	81.7	6	8	1.7	6.1	8.5	22	12.3	17.5	67.3	52.4	
20	76	83.9	5	8	1.6	5.7	7.9	36.2	10.5	14.3	55.5	58.7	
21	78	86.4	5	8	1.9	5.9	10.1	28.8	11.1	11.1	46.2	65.1	
22	78.1	88	4	8	0.47	1.6	8.8	24.8	7	11.1	41.5	65.1	
23	78.7	89	3	7	0.2	4.8	9.4	21.5	3.5	11.1	33.3	71.4	
24	78.9	89.6	3	6	0.68	1.6	9.7	22.2	7	11.1	38	77.8	

Data for weather described monthly mean values derived from daily records in 2016 and 2017 (Table 3, Table 4).

**Table 3:** Weather report of Federal University of Agriculture Abeokuta, for 2016.

		Jan	Feb	Mar	Apr	May	Jun	Jul	Aug	Sept	Oct	Nov	Dec
Rainfall total (mm)		32.0	0.0	150.3	68.2	226.2	150.5	65.2	63.6	229.0	155.4	5.9	0.0
Sunshine (h)		4.0	3.3	2.0	6.3	5.1	4.0	2.8	1.95	2.7	4.9	5.5	5.5
Relative humidity (%)		56.2	56.7	59.1	63.1	73.6	72	72.7	72.8	68.9	65.3	65.3	56.6
Evaporation (mm)		4.0	3.6	3.9	4.5	2.4	2.0	1.5	2.2	1.2	2.4	4.2	6.5
Soil temp. (°C)	10 cm	27.1	29.8	30.5	29.8	28.8	28.0	25.9	26.1	26.6	27.4	28.5	29.3
	20 cm	28.1	30.1	30.7	30.0	29.1	28.2	27.1	26.5	26.8	27.6	28.7	29.7
Mean temp. max/min. (°C)		28.1	30.3	29.5	29.2	29.0	26.7	26.3	25.7	26.9	27.6	28.0	22.5

Sourced from Department of Agrometeorology and Water Management, Federal University of Agriculture Abeokuta.

**Table 4:** Weather report of Federal University of Agriculture Abeokuta, for 2017.

		Jan	Feb	Mar	Apr	May	Jun	Jul	Aug	Sept	Oct	Nov	Dec
Rainfall total (mm)		15.9	0.0	34.3	112.8	146	111	156.1	90.5	50.0	92.2	45.6	0.0
Sunshine (h)		4.4	4.2	6.1	5.6	5.2	4.3	2.1	1.3	2.1	4.4	5.8	4.2
Relative humidity (%)		60.6	55.3	59.2	63.2	68.9	73.8	74.5	77.4	69.1	72.8	65.7	69.2
Evaporation (mm)		4.5	5.5	5.4	4.4	2.4	1.4	1.4	1.6	1.7	2.7	3.5	3.9
Soil temp. (°C)	10 cm	29.6	29.6	31.6	29.8	28.2	28.0	26.9	26.3	26.4	27.2	28.4	28.4
	20 cm	29.5	30.8	32.1	29.3	28.5	28.3	27.2	26.4	26.7	27.7	28.8	28.8
Mean temp. max/min. (°C)		28.1	30.3	29.5	29.2	29.0	26.7	26.3	25.7	26.9	27.6	28.0	22.5

Sourced from Department of Agrometeorology and Water Management, Federal University of Agriculture Abeokuta.

Commercial loss of rhizomes to rot disease of *C. amada* Plant:

$$\mathrm{Percentage\backslash quantity}\;(\mathrm{commercial})\;\mathrm{loss\backslash for}\;2016=\;\frac{\mathrm{WH}-\mathrm{WC}}{\mathrm{WH}}\times 100=\frac{36.60\mathrm{Kg}-35.41\mathrm{Kg}}{36.60\mathrm{Kg}}\times 100=\frac{1.19}{36.60}\times 100=3.25\%$$

$$\mathrm{Percentage\backslash quality}\;\left(\mathrm{commercial}\right)\;\mathrm{loss\backslash for}\;2017=\;\frac{\mathrm{WH}-\mathrm{WC}}{\mathrm{WH}}\times 100=\;\frac{44.02\mathrm{Kg}-42.69\mathrm{Kg}}{44.02\mathrm{Kg}}\times 100=\;\frac{1.33}{44.02}\times 100=3.02\%$$

Where,

Weight of harvested rhizomes = WH in kilogrammes
Weight of clean rhizomes = WC in kilogrammes

## 2 Experimental design, materials and methods

Survey cultivation (on 20th June, 2016 and 11th April, 2017) of *C. amada* was done according to a quasi experimental design described in https://doi.org/10.1016/j.cpb.2018.10.001 [1].

Pictorial data showing diseases symptom were gotten by photograph on fields of population of the Mango ginger plants.

Data of fungi associated with diseases of Mango ginger were gotten from field sample collections of diseased organs and laboratory isolations of associated fungi, from diseased tissues as described by Narayanasamy [3]. Associated organisms were identified with literatures and by expert mycologists.

Data of height of plants and number of leaves were gotten by weekly measurement with metre rule and counting, respectively, from the first week to the twenty-fourth week of emergence of sprouts. Correspondingly, data for spot disease incidence and blight disease incidence were derived from counting and calculation, while spot disease severity and blight disease severity were derived by disease assessment scale and calculation as applicable in https://doi.org/10.1016/j.cpb.2018.10.001 [1]

Data of weather for the two years were sourced from Department of Agrometeorology and Water Management, Federal University of Agriculture Abeokuta (Table 5).

**Table 5:** Field plant disease severities were assessed on the disease assessment scale used by Jaydeep et al. [2], showing different ratings on 0–9 scale.

Ratings	Description
0	No symptom
1	Disease covering less than 1% leaf area
3	Disease covering 1–10% leaf area
5	Disease covering 11–25% leaf area
7	Disease covering 26–50% leaf area
9	Disease covering greater than 50% leaf area
