# Supplementary material for: Data for the description of fungal diseases and agronomic parameters of Mango ginger (Curcuma amada Roxb.)
Source: Data Brief. 2018 Nov 16;21:2536–42. doi: 10.1016/j.dib.2018.11.065 (PMC6288459; doi:10.1016/j.dib.2018.11.065)
Supplement: Supplementary file 1 — Supplementary material [file mmc1.doc]

**Conflict of Interest**

The data in this manuscript was sourced through research by me, the author. The manuscript has been revised according to the reviewer’s direction and is titled: Data for the Description of Fungal Diseases and Agronomic Parameters of Mango ginger (*Curcuma amada* Roxb.).

The manuscript has not been submitted to, nor been under review at another journal or other publishing body, and I have no affiliation with any organization with a direct or indirect financial interest in the subject matter discussed in the manuscript.

Thank you.

Ayodele Victor

11 November, 2018
